# A simulation study to improve calcium intake through wheat flour fortification

**DOI:** 10.1017/S1368980024001228

**Published:** 2024-06-03

**Authors:** Gabriela Cormick, Iris B Romero, María B Puchulu, Surya M Perez, Miriam Sosa, Lorena Garitta, Eliana Elizagoyen, Maria Fernanda Gugole, José M Belizán, Natalia Matamoros, Luz Gibbons

**Affiliations:** 1 Centro de Investigaciones en Epidemiología y Salud Pública (CIESP - IECS), Consejo Nacional de Investigaciones Científicas y Técnicas (CONICET), Buenos Aires, Argentina; 2 Instituto de Efectividad Clínica y Sanitaria (IECS – CONICET), Buenos Aires, Argentina; 3 Universidad Nacional de La Matanza, San Justo, Argentina; 4 Departamento de Ciencias Fisiológicas, Universidad de Buenos Aires, Facultad de Medicina, Buenos Aires, Argentina; 5 Departamento de Evaluación Sensorial de Alimentos (DESA), Instituto Superior Experimental de Tecnología Alimentaria (ISETA), Buenos Aires, Argentina; 6 Consejo Nacional de Investigaciones Científicas y Técnicas (CONICET), Buenos Aires, Argentina; 7 Comisión de Investigaciones científicas de la provincia de Bs As (CIC), Buenos Aires, Argentina; 8 Instituto de Desarrollo E Investigaciones Pediátricas “Prof. Dr. Fernando E. Viteri” Hospital de Niños “Sor María Ludovica de La Plata (IDIP), Ministerio de Salud/Comisión de Investigaciones Científicas de La Provincia de Buenos Aires, La Plata, Argentina

**Keywords:** Calcium, Micronutrient, Fortification, Flour, Inadequate intake

## Abstract

**Objective:**

To simulate the impact on calcium intake – effectiveness and safety – of fortifying wheat flour with 200, 400 and 500 mg of calcium per 100 g of flour.

**Design:**

Secondary analysis of cross-sectional data collected through repeated 24 h dietary recalls using the Iowa State University Intake Modelling, Assessment and Planning Program.

**Setting:**

Urban cities in the National Health and Nutrition Survey of Argentina (ENNyS 2018–2019).

**Participants:**

21 358 participants, including children, adolescents and adults.

**Results:**

Most individuals in all age groups reported consuming wheat flour. The prevalence of low calcium intake was above 80 % in individuals older than 9 years. Simulating the fortification of 500 mg of calcium per 100 g of wheat flour showed that the prevalence of low calcium intake could be reduced by more than 40 percentage points in girls and women aged 19 to less than 51 years and boys and men aged 4 to less than 71 years, while it remained above 65 % in older ages. The percentages above the upper intake level remained below 1·5 % in all age groups.

**Conclusions:**

Calcium flour fortification could be further explored to improve calcium intake. Subnational simulations could be performed to identify groups that might not be reached by this strategy that could be explored in Argentina. This analysis could be used to advocate for a strategy to fortify wheat flour.

Calcium is the most abundant mineral in the human body with more than 99 % stored in bone tissue as hydroxyapatite, a key structural function of the skeletal system^(1)^. The remaining 1 % is found in soft tissues and in fluids, such as blood^(1)^. Calcium is involved in several vital functions, including blood coagulation, cardiac and skeletal muscle contraction, neuronal signalling, secretory activity, apoptosis, immune response, cell differentiation and enzyme activation^(1–3)^. All calcium necessary for growth and replenishment of daily losses must be supplied via food sources as this essential nutrient is not synthesised by the human body^(3)^. Unfortunately, there are no practical population-level markers of calcium status. Variations in calcium intake are typically not reflected in calcium serum levels^(3)^. Serum calcium concentration is tightly maintained at levels around 1·0–1·2 mmol/l by homeostatic mechanisms that regulate renal excretion and reabsorption, intestinal calcium absorption and bone formation^(3)^. Since there is a lack of reliable biochemical marker for calcium status, calcium adequacy is usually measured by comparing population dietary intakes with dietary reference values^(4)^. Calcium dietary reference values vary by age, gender and life stages (pregnancy and lactation) and also by reference guidelines^(3)^. The FAO and WHO suggest a recommended nutrient intake of 1000 mg calcium per day for young adults and 1300 mg/d for men over 65 years and for postmenopausal women^(4)^. The US Institute of Medicine (IOM) suggests a RDA of 1000 mg/d for most adults but 1200 mg/d for postmenopausal women^(5)^. According to data from food balance sheets, approximately half of the world’s population has inadequate access to appropriate foods to cover their dietary calcium needs^(6)^. A systematic review performed by the International Osteoporosis Foundation Calcium Steering Committee compiled available data on average national dietary calcium intake around the globe and found that calcium intake is low (averaging < 400 mg/d) in many countries of Southeast Asia and around 400–600 mg/d in countries of South America, including Argentina^(7)^. The study revealed a health inequity as the lowest calcium availability from foods and lowest calcium intakes are largely found in low- and middle-income countries of Asia, Africa and Latin America^(8,9)^.

Randomised controlled trials performed on children, adolescents, pregnant women, women of reproductive age and postmenopausal women have shown the impact of improving calcium intake through calcium supplementation and calcium-fortified foods^(10)^. The impact was observed on health outcomes including height, bone mineral density and perinatal health^(10)^. Hypertensive disorders of pregnancy cause around 50 000 maternal deaths and 500 000 neonatal deaths annually worldwide, making them one of the leading obstetric causes of maternal mortality globally^(11,12)^. Calcium supplementation reduces the occurrence of hypertensive disorders of pregnancy and halves the occurrence of pre-eclampsia in populations with low calcium intake^(13)^. Maternal mortality ratio in Argentina is forty-five deaths per 100 000 live births with no clear decline in the last 10 years^(14)^. Hypertensive disorders of pregnancy represent the main obstetric cause of maternal mortality in Argentina, and calcium is one of the most deficient micronutrients in the population, with little changes since 2005^(14,15)^.

The WHO recommends exploring calcium fortification of staple foods in populations with low calcium intake as it is an important public health intervention for the prevention of pre-eclampsia, as well as having additional benefits for the general population^(16)^. Food fortification is an effective strategy to improve micronutrient intake^(17)^. Calcium may be added to certain staple foods, such as wheat flour, maize flour or cornmeal, rice and dairy products^(9)^. However, few countries have official regulations and/or food standards for calcium fortification^(9,17)^. Different analyses have modelled the impact of water and flour calcium fortification in low- and middle-income countries and high-income countries^(15,18–20)^.

A recent analysis from the second Nutrition and Health National Survey in Argentina (abbreviated as ENNyS2 in Spanish) found that the prevalence of low calcium intake was as high as 88 % in girls and women^(21)^.

The objective of this study was to simulate the impact – effectiveness and safety – of fortifying wheat flour with 200, 400 and 500 mg of calcium per 100 g of flour using the ENNyS2 performed in Argentina between 2018 and 2019^(22)^. This would be the first step towards designing a strategy to fortify wheat flour with calcium at a population level.

## Materials and methods

This study is a simulation to assess the impact of wheat flour fortified at different calcium levels on overall calcium intake. We estimated the calcium intake in different age groups and then simulated the impact of fortifying white wheat flour with 200, 400 and 500 mg of calcium per 100 g of flour. This analysis was performed to determine which calcium level would be most effective in reducing low calcium intake in the population, defined as the proportion of individuals in the group with usual calcium intake below the age-specific estimated average requirement (EAR), while minimising those at risk of excess in calcium intake, defined as the proportion of individuals who exceeded the tolerable upper intake level (UL).

### Population

Data were obtained from the ENNyS2, a cross-sectional survey carried out in urban areas by the Ministry of Health and Social Development of Argentina between 2018 and 2019, designed to extrapolate the results to the whole urban population of Argentina^(22)^. Participants were selected using a probabilistic complex sample design including small and large cities from all provinces of Argentina. A total of 21 358 participants, 5763 infants, 8228 children and adolescents and 7367 adults were included in the sample. The ENNyS required 1200 participants by age and country region to be representative at the regional level. The final sample was reached for all regions except greater Buenos Aires, which is the most populated region in the country. Age group representativeness was reached by all age groups except children less than 2 years old^(22)^.

### Dietary assessment

Dietary assessment was performed in person by trained dietitians^(22)^. Data were collected using a standardised multiple-pass 24 h diet recall designed to capture all foods, beverages, supplements and medicines consumed by participants on the previous day. A repeated 24 h dietary recall was performed on 20 % of the population at least 48 h after the first dietary recall. A Digital Photographic Food Atlas was designed to estimate portion sizes. Primarily, we used the local food chemical composition table (Sistema de Análisis y Registro de Alimentos 2, SARA2 in Spanish abbreviation) to estimate nutrient intake^(23)^. This food chemical composition table is a compilation of data from local sources such as ARGENFOODS. The SARA2 methodology describes that when local data were not available, the chemical composition of similar foods from other countries’ chemical composition tables, like the UK and USA, was added to the local database^(24–26)^. In this way, if a food item is fortified in one composition table, but not in Argentina, it was not used. The SARA2 presents data on 1105 food items including mandatory and voluntary fortified foods and beverages as well as brand-specific foods and beverages available in the local market. Data are presented in twenty-six food groups and thirty-nine food components including energy, water and macro- and micronutrients^(23)^. We used food labels when a food item was not available in the database.

### Analysis

All analyses were adjusted for the complex sample design weight. We used the Intake Modelling Assessment Program developed by Iowa State University (IOWA), a computer program that allows running different simulation scenarios of nutrient intake using dietary intake information of population age groups. We first calculated the calcium intake distribution of one single day and calculated the day-to-day variability of calcium intake using the repeated 24 h dietary recalls. This calcium intake distribution included calcium-containing foods, beverages, supplements and medicines reported in the ENNyS. We then adjusted the calcium intake distribution with the day-to-day variability to obtain an estimated distribution of the usual calcium intake.

Afterwards, we calculated the baseline prevalence of inadequate calcium intake as the proportion of individuals in the group with usual calcium intake below the age-specific EAR and the risk of excess as the proportion of individuals with usual calcium intakes above the age-specific UL. We used the default harmonised dietary reference values that the Intake Modelling Assessment Program assigns to each population group. These reference values are mainly a compilation of EAR and UL from IOM’s RDA and recommended nutrient intake from the FAO/WHO tables^(27)^. The calcium EAR and UL values used for this analysis are presented in Table 1.

**Table 1:** Dietary reference values, white wheat flour and calcium intake, percentage of the population below the estimated average requirement (EAR) and percentage of the population over the recommended upper intake level (UL) for calcium intake before and after the simulation of wheat flour fortification with 200, 400 and 500 mg of calcium per 100 g of wheat flour using dietary intake from the ENNyS2 carried out in Argentina (2018–2019)

Population group	*n*	Repeated recall	EAR	UL	White flour intake			Calcium intake before simulations			
					%	Mean (g)	SD	Mean (mg)	sd	<EAR * (%)	>UL ** (%)
Children											
1 <= age < 4	4055	924	400	2500	95·6	42·2	36·3	722	379	20·3	0·1
4 <= age < 9	2622	575	640	2500	98·9	77·1	52·5	652	288	53·2	0·0
Boys and adult males											
9 <= age < 14	1283	300	1100	3000	99·0	103·1	69·8	606	285	94·4	0·0
14 <= age < 19	1039	241	1100	3000	98·9	114·1	80·1	618	307	92·5	0·0
19 <= age < 31	884	195	800	2500	97·7	110·0	74·3	578	286	80·6	0·0
31 <= age < 51	1069	234	800	2500	97·2	101·8	77·2	560	197	88·4	0·0
51 <= age < 71	781	169	800	2000	96·3	79·6	56·3	511	245	88·1	0·0
71 <= age	243	52	1000	2000	97·5	67·9	50·8	545	305	92·2	0·3
Girls and not pregnant adult females											
9 <= age < 14	1153	279	1100	3000	99·1	83·9	57·5	558	243	97·3	0·0
14 <= age < 19	1071	234	1100	3000	97·1	87·3	64·8	524	229	98·0	0·0
19 <= age < 31	1189	255	800	2500	97·3	85·1	61·0	502	210	91·0	0·0
31 <= age < 51	1561	366	800	2500	95·9	68·7	54·7	502	231	89·4	0·0
51 <= age < 71	993	221	1000	2000	96·8	59·4	46·4	500	230	96·5	0·0
71 <= age	365	94	1000	2000	97·8	55·4	40·7	557	265	93·3	0·0

Population group	Calcium intake after simulations														
	Fortified with 200 mg in 100 g of flour					Fortified with 400 mg in 100 g of flour					Fortified with 500 mg in 100 g of flour				
	Mean (mg)	sd	<EAR (%)	>UL (%)	% *Reduction	Mean (mg)	sd	<EAR (%)	>UL (%)	% *** Reduction	Mean (mg)	sd	<EAR (%)	>UL (%)	% *Reduction
Children															
1 <= age < 4	808	383	12·4	0·1	−7·9	894	394	7·5	0·2	−12·8	937	402	5·8	0·2	−14·5
4 <= age < 9	806	294	31·0	0·0	−22·2	961	316	14·6	0·0	−38·6	1038	329	9·6	0·0	−43·6
Boys and adult males															
9 <= age < 14	812	316	82·8	0·0	−11·6	1019	358	63·1	0·0	−31·3	1124	384	52·1	0·0	−42·3
14 <= age < 19	851	329	79·9	0·0	−12·6	1084	375	56·8	0·0	−35·7	1200	402	44·7	0·0	−47·8
19 <= age < 31	802	331	55·5	0·0	−25·1	1027	388	30·5	0·2	−50·1	1139	421	21·9	0·5	−58·7
31 <= age < 51	763	210	61·6	0·0	−26·8	965	258	27·8	0·0	−60·6	1066	287	17·5	0·0	−70·9
51 <= age < 71	672	258	73·8	0·0	−14·3	833	281	50·7	0·2	−37·4	913	296	39·1	0·3	−49·0
71 <= age	686	326	85,7	0·5	−6·5	825	353	74·7	0·9	−17·5	895	372	67·9	1·3	−24·3
Girls and not pregnant adult females															
9 <= age < 14	729	256	91·6	0·0	−5·7	900	286	77·6	0·0	−19·7	984	304	68·2	0·0	−29·1
14 <= age < 19	701	261	92·3	0·0	−5·7	877	312	78·4	0·0	−19·6	966	342	69·2	0·0	−28·8
19 <= age < 31	675	244	74·0	0·0	−17·0	848	274	47·8	0·2	−43·2	935	298	35·9	0·0	−55·1
31 <= age < 51	644	249	76·8	0·0	−12·6	785	277	57·6	0·0	−31·8	856	292	47·4	0·0	−42·0
51 <= age < 71	622	250	92·1	0·0	−4·4	743	277	83·7	0·1	−12·8	804	294	77·8	0·2	−18·7
71 <= age	666	274	87·9	0·0	−5·4	776	292	79·1	0·0	−14·2	831	305	73·6	0·2	−19·7

We then performed a distribution of wheat flour and calcium intake and estimated the calcium required to decrease the population level of calcium inadequacy without exceeding the recommended calcium UL for each population group^(27)^. We calculated the ‘initial gap’ defined as the estimated amount of calcium in flour to achieve the target prevalence of inadequate intakes. The initial gap was calculated as the difference in mg per day between the EAR for the target population group and the usual calcium intake for that group.

To perform the calcium fortification simulation, we used the ENNyS database to identify foods and beverages such as pizza, bread, cakes and milkshakes that might contain white wheat flour. We then calculated the percentage of white wheat flour in each consumed item to obtain the total wheat flour intake in grams. We used local recipes obtained from the Nutritionists Federation of Argentina (FAGRAN), websites, labels and documents showing standardised recipes such as the UK bread and flour regulations^(28)^. When an item contained a mix of flours, we calculated the amount of wheat flour in that item.

Finally, using the Intake Modelling Assessment Program, we estimated the potential impact of different calcium fortification levels in wheat flour. To assess the impact on inadequate calcium intake and the risk of excess, we estimated the adjusted calcium intake distributions for each age group after simulating the addition of 200, 400 and 500 mg of calcium per 100 g of wheat flour. We measured the effectiveness of each fortification level, as the percentage of individuals below the calcium EAR and measured safety or risk of excess as the percentage of individuals exceeding the calcium recommended UL of their corresponding age-specific population subgroup^(27)^.

## Results

The number of participants, mean wheat white flour intake, mean calcium intake before and after simulation, prevalence of low calcium intake before and after simulation, and percentage of individuals exceeding the UL before and after simulation are presented by age–sex categories in Table 1. Flour and calcium intake by country region is reported in Table 2. The prevalence of low calcium intake, defined as the proportion of individuals in the group with usual calcium intake below the age-specific EAR, was above 80 %, in age groups older than 9 years (Table 1). Only 2·4 % of participants reported taking any kind of supplements, and less than 0·1 % of participants reported taking calcium supplements.

**Table 2:** White wheat flour and calcium intake by country region, National Nutrition and Health Survey (ENNyS 2) carried out in Argentina (2018–2019)

	GBA						Centro						NEA					
	At least some flour Intake		Flour intake		Calcium intake		At least some flour Intake		Flour intake		Calcium intake		At least some flour Intake		Flour intake		Calcium intake	
Population group	*n*/*N*	%	Mean	sd	Mean	sd	*n*/*N*	%	Mean	sd	Mean	sd	*n*/*N*	%	Mean	sd	Mean	sd
1 <= age < 4	586/615	95	47	40	686	408	678/695	98	47	38	755	459	646/696	93	46	41	774	433
4 <= age < 9	386/391	99	73	45	670	373	475/478	99	84	54	691	391	386/395	98	90	64	734	430
Male: 9 <= age < 14	177/179	99	98	63	654	430	208/211	99	116	72	735	478	207/211	98	121	93	641	357
Male: 14 <= age < 19	138/141	98	116	80	743	528	167/171	98	118	84	765	525	188/192	98	135	104	578	380
Male: 19 <= age < 31	105/108	97	113	79	601	388	123/125	98	104	66	728	528	173/177	98	127	88	628	405
Male: 31 <= age < 51	139/150	93	86	64	626	387	180/186	97	109	78	630	420	153/159	96	126	115	562	372
Male: 51 <= age < 71	87/91	96	75	53	653	389	133/141	94	79	63	607	406	116/123	94	92	63	524	390
Male: 71 >= age	29/29	100	66	41	483	273	54/54	100	63	45	652	552	41/45	91	80	60	545	294
Female: 9 <= age < 14	149/150	99	79	51	571	335	221/221	100	89	52	659	379	172/175	98	103	85	596	355
Female: 14 <= age < 19	125/129	97	91	70	605	487	172/181	95	91	69	582	396	184/194	95	95	78	520	342
Female: 19 <= age < 31	128/134	96	85	63	493	335	175/178	98	88	55	603	395	207/217	95	96	73	535	321
Female: 31 <= age < 51	186/195	95	67	54	563	353	238/254	94	73	58	578	403	243/254	96	74	65	521	341
Female: 51 <= age < 71	121/125	97	55	42	588	364	186/190	98	58	43	505	353	152/163	93	65	61	536	318
Female: 71 >= age	37/38	97	47	37	580	298	82/83	99	57	42	596	364	67/69	97	62	45	630	343

	NOA						Cuyo						Patagonia					
	At least some flour intake		Flour Intake		Calcium intake, mean		At least some flour intake		Flour intake		Calcium intake		At least some flour intake		Flour intake		Calcium intake	
Population group	*n*/*N*	%	Mean	sd	Mean	sd	*n*/*N*	%	Mean	sd	Mean	sd	*n*/*N*	%	Mean	sd	Mean	sd
1 <= age < 4	706/759	93	37	34	728	455	639/677	94	36	31	700	467	584/613	95	40	31	641	375
4 <= age < 9	474/485	98	72	46	576	410	426/429	99	69	45	594	385	437/444	98	77	58	641	380
Male: 9 <= age < 14	236/237	100	102	58	494	387	222/225	99	92	60	549	411	217/220	99	91	63	632	416
Male: 14 <= age < 19	194/194	100	109	71	527	401	205/205	100	108	65	589	442	133/136	98	95	63	592	432
Male: 19 <= age < 31	182/187	97	103	67	525	406	167/171	98	110	69	492	354	110/116	95	100	72	531	360
Male: 31 <= age < 51	188/192	98	103	68	491	368	206/210	98	92	59	495	373	163/172	95	96	67	597	387
Male: 51 <= age < 71	123/126	98	87	61	428	368	170/175	97	76	47	439	298	120/125	96	69	48	514	338
Male: 71 >= age	29/29	100	90	63	410	291	41/42	98	58	32	486	376	42/44	95	58	54	562	565
Female: 9 <= age < 14	206/211	98	77	45	472	344	205/208	99	76	48	494	324	186/188	99	80	55	573	400
Female: 14 <= age < 19	199/207	96	91	58	450	324	183/190	96	80	54	464	348	166/170	98	75	58	520	370
Female: 19 <= age < 31	241/246	98	89	63	445	312	226/237	95	77	53	430	322	169/177	95	75	53	507	365
Female: 31 <= age < 51	270/282	96	73	59	428	307	274/283	97	64	42	435	305	276/293	94	62	48	513	332
Female: 51 <= age < 71	184/187	98	61	45	421	338	156/164	95	58	44	439	307	157/165	95	59	40	519	311
Female: 71 >= age	53/56	95	63	45	441	331	64/64	100	55	37	507	349	53/55	96	43	32	523	330

GBA: Greater Buenos Aires; NEA: Argentine Northeast region; NOA: Argentine Northwest region.

Wheat flour intake was reported by more than 95 % of individuals in all age groups (Table 1) and by more than 90 % of individuals in all age groups from each region of the country (Table 2).

The highest levels of wheat flour intake were found in those aged 9–51 years. Mean wheat flour intake ranged from 101·8 to 110·0 g/d in men aged 19–51 years, and wheat flour intake was lower in women. Women aged 51 years and older showed the lowest wheat flour intake of the whole adult population with values ranging from 55·4 to 59·4 g/d (Table 1).

Changes in calcium intake after simulating wheat flour fortification with different calcium levels are presented in Table 1. The simulation with a fortification level of 200 mg of calcium per 100 g of flour showed that the prevalence of low calcium intake could be reduced from 20·3 % to 12·4 % in children aged 1 to less than 4 years and from 53·2  % to 31·0 % in children aged 4 to less than 9 years. In girls aged 9 to less than 14 years, the prevalence of low calcium intake was slightly reduced, from 97·3 % to 91·6 %, whereas in boys of the same age, it could be reduced from 94·4 % to 82·8 %. The prevalence of low calcium intake remained around 80 % for adolecent boys and around 90 % for adolecent girls after simulating a fortification of 200 mg of calcium per 100 g of flour (Table 1). A greater impact was observed in men. The prevalence of low calcium intake could be reduced from 80·6 % to 55·5 % in those aged 19 to less than 31 years (representing a reduction of 25·1 percentage points), from 88·4 % to 61·6 % in those aged 31 to less than 51 years (representing a reduction of 26·8 percentage points), from 88·1 % to 73·8 % in those 51 to less than 71 years (representing a reduction of 14·3 percentage points) (Table 1). The results of the simulation showed a smaller impact in women aged 19 to less than 71 and for men and women aged 71 years or older. After the simulation of a wheat flour fortification with 200 mg of calcium per 100 g of wheat flour, none of the age groups studied showed more than 0·5 % above the UL which was our cut off for safety (Table 1).

The simulation of a wheat flour fortification with 400 mg of calcium per 100 g of wheat flour allowed greater reductions in inadequate calcium intake. The prevalence of low calcium intake could be reduced by more than 30 percentage points in girls and women aged 19 to less than 51 years and boys and men aged 9 to less than 71 years. The prevalence of low calcium intake remained above 70 % in older ages. With a fortification of 400 mg of calcium per 100 g of flour, percentages above the UL remained at 0·2 % or lower in all age groups, except for men aged 71 years or older where it reached 0·9 %.

The simulation of wheat flour fortification with 500 mg of calcium per 100 g of wheat flour allowed even greater reductions in the percentage of inadequate calcium intake. The prevalence of low calcium intake could be reduced by more than 40 percentage points in girls and women aged 19 to less than 51 years and boys and men aged 9 to less than 71 years. The prevalence of low calcium intake remained high in older ages. With a fortification of 500 mg of calcium per 100 g of flour, percentages above the UL remained at 0·5 % or lower in all age groups, except for men aged 71 years or older where it reached 1·3 %.

**Fig. 1:**
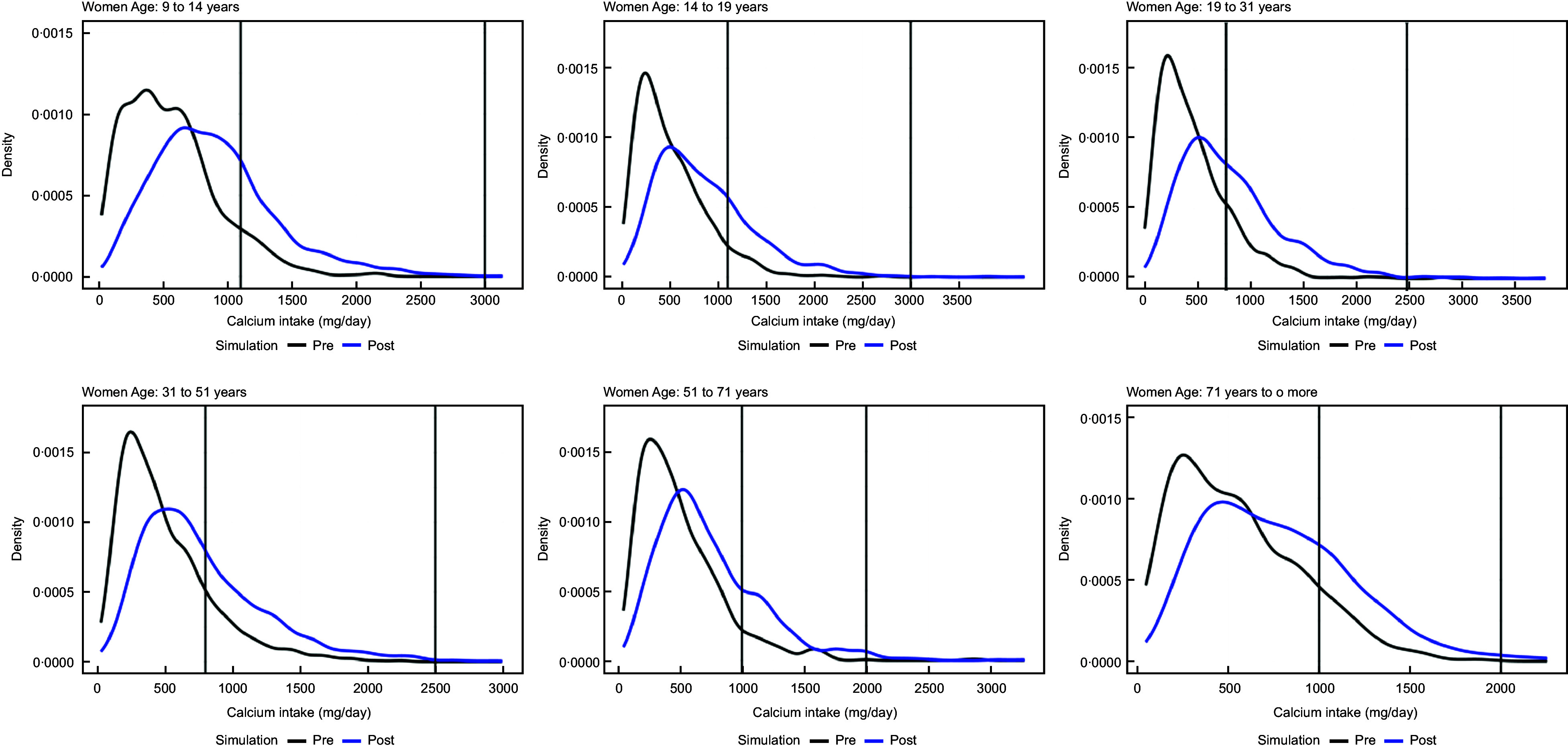
Distribution of calcium intake in women after simulating a fortification with 400 mg of calcium per 100 g of wheat flour

**Fig. 2:**
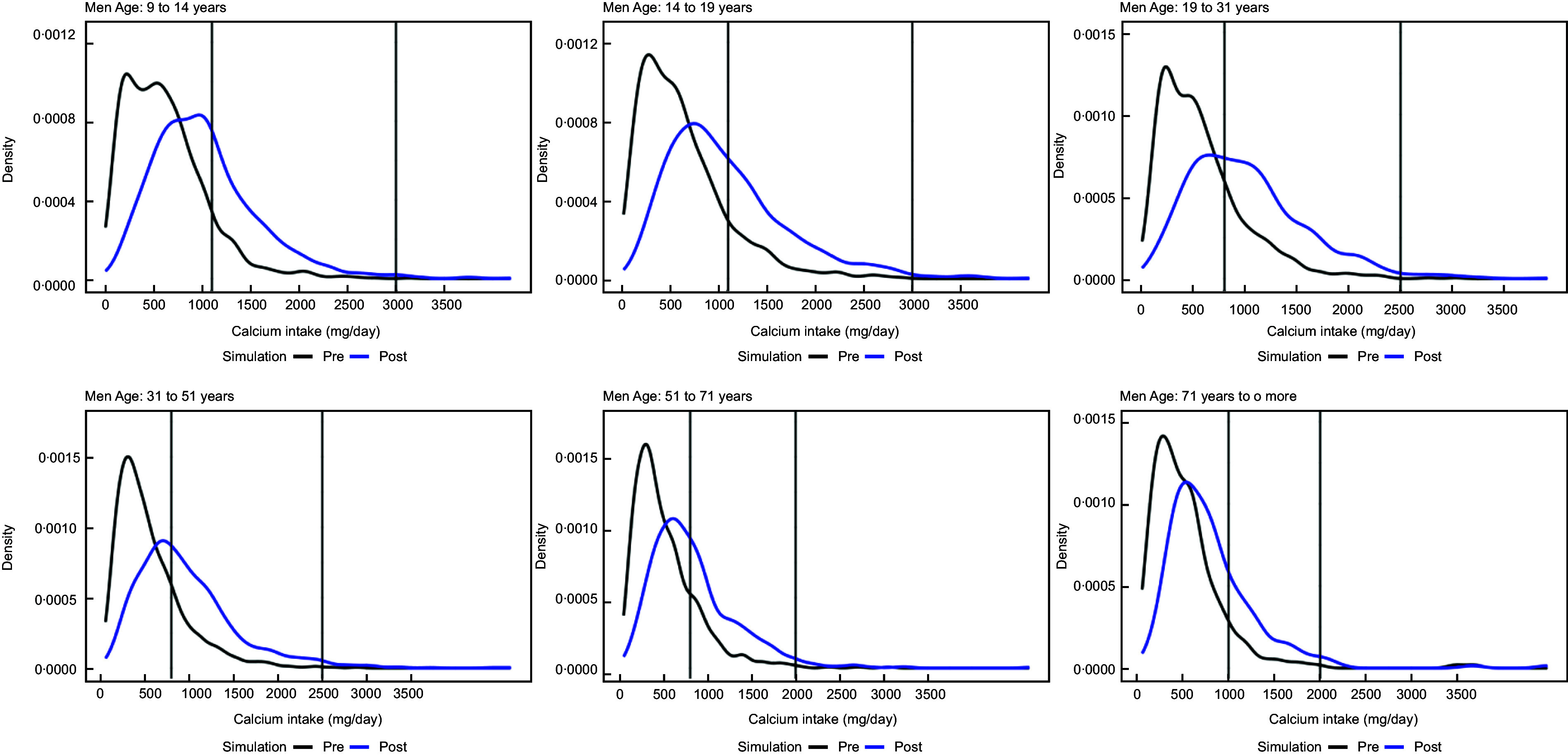
Distribution of calcium intake in men after simulating a fortification with 400 mg of calcium per 100 g of wheat flour

The original distribution of calcium intake and the distribution of calcium intake after simulating a fortification with 400 mg of calcium per 100 g of wheat flour for women and men are shown in Figs. 1 and 2, respectively.

## Discussion

In this simulation, we show that it is effective and safe to improve calcium intake in Argentina through fortifying wheat flour at levels of 200, 400 and 500 mg of elemental calcium per 100 g of wheat flour. With a fortification of 200 mg of calcium per 100 g of flour, the prevalence of low calcium intake would be reduced by more than 20 percentage points in men aged 19 to less than 51. At this fortification level, it was more difficult to reduce the prevalence of low calcium intake in individuals aged 9 to less than 19 years, women and men older than 71 years. The simulation of a wheat flour fortification with 400 and 500 mg of calcium per 100 g of flour showed reductions in the prevalence of low calcium intake of 40 percentage points or more in children aged 4 to less than 9 years, women aged 19 to less than 31 years and men aged 19 to less than 71 years.

Our analysis using the ENNyS2 shows that the prevalence of low calcium intake in Argentina is high, reaching above 80 % in all groups 9 years or older^(22)^. The calcium intake we report for women and children in this analysis is similar to a previous analysis that used data from the first national survey in Argentina (ENNyS1), indicating that calcium intake remains low, with minimal changes since 2005^(18)^. In this analysis, we show that the prevalence of low calcium intake is also high in adolescent boys and adult men, both of which were groups not included in the ENNyS1^(18)^. Adolescent boys and adult men are important to take into consideration as they usually have a higher food intake than the rest of the population and are prone to reach the UL more easily with the introduction of fortified foods. Groups with higher food intake may limit the maximum level that can be recommended for a large-scale food fortification policy, which needs to be effective for the most vulnerable populations, yet safe for the whole population^(4)^.

The UL is the highest level of calcium intake that is likely to pose no risk of adverse health effects^(29)^. In our analysis, the risk of exceeding the UL, defined as the proportion of individuals in the group with usual calcium intake above the age-specific UL, started to increase in men aged 71 years or more with the simulation of a fortification level with 400 mg of calcium per 100 g of wheat flour; however, it only reached 0·9 %. The simulation of wheat flour fortification with 500 mg of calcium per 100 g of flour produced further reductions in the prevalence of low calcium intake, while the risk of exceeding the UL remained low, reaching 1·3 % only in men aged 71 years or more. In Argentina, the use of micronutrient supplements is low, so the risk of exceeding the UL is low. However, even in countries like the USA, where micronutrient supplement intake is common, the risk of exceeding the calcium UL was also reported to be low, at around 3 %^(30)^. In our simulations, we used the default Intake Modelling Assessment Program harmonised dietary reference values, with an UL of 2000 mg of calcium a day for those aged 71 years or older. This level is still under discussion, and it is considerably lower than the 2500 mg of calcium a day used by other standards^(31)^. For individuals aged 71 years or older, the European Food Safety Authority panel recommends 2500 mg of calcium a day, the same UL as younger groups, as data from population-based studies indicate that calcium intake can be close to this level without posing any health risks^(31)^. The European Food Safety Authority panel based their recommendation on the results of long-term clinical trials in which individuals received a daily supplement containing 2500 mg of calcium, besides their habitual food calcium intake^(31)^. These clinical trials found no evidence of increased risk of hypercalciuria, nephrolithiasis, CVD or prostate cancer^(31)^. Running this simulation with a higher UL of 2500 mg of calcium a day would produce a fortification level that yields greater reductions of low calcium intake without increasing older adult’s risk of exceeding the UL.

Our analysis is limited to the population data included in ENNyS2, the latest national survey available in Argentina. The ENNyS2 includes only the urban population, as most of the population of Argentina lives in urban areas (92·5 %). Further studies should estimate the impact of wheat flour fortification in rural populations^(32)^. Results from children under 2 years of age and the region of Greater Buenos Aires should be taken with caution and confirmed by further studies as samples did not reach representativeness.

Low calcium intake is common worldwide; however, the percentage of the population with low calcium intake differs widely between age groups and populations^(7)^. In our previous simulation analysis using a fortification of 156 mg of calcium per 100 g of flour, we showed reductions in the percentage of population with low calcium intake in low- and middle-income countries such as Argentina, Uganda and Zambia. However, in high-income countries, a population-based strategy could be limited by the risk of men exceeding the calcium intake UL at this fortification level^(18)^. Despite having an average calcium intake higher than in low- and middle-income countries, data from the US National Health and Nutrition Examination Survey (NHANES) 2009–2010 indicated that 42 % of Americans did not meet their IOM-EAR for calcium^(33,34)^. A study using a large national survey performed in Canada modelled a mandatory calcium fortification of food products with levels from 55 to 165 mg of calcium per serving. The results show that the fortification would be effective in reducing the prevalence of low calcium intake in Canada; however, despite being beneficial for most population age groups, the risk of excess would increase in men, reaching 7 % of individuals above the UL^(20)^. On the other hand, evidence from the UK’s long-term experience of mandatory calcium fortification with levels between 94 and 156 mg of elemental calcium per 100 g of white wheat flour demonstrated that calcium fortification increases calcium intake and has a positive impact on bone health with no adverse effects^(35)^. It is estimated that calcium-fortified wheat flour contributes to 13–14 % of the total calcium intake in the UK, and without this policy, an additional 10–12 % of adolescents would not meet the recommended intake^(17)^. Since the implementation of mandatory calcium fortification of wheat flour in the UK, few countries have adopted mandatory or voluntary wheat flour fortification, mainly with levels between 125 and 312·5 mg of calcium per 100 g of flour^(17)^. In Germany, calcium fortification of bread was shown to improve bone mineralisation, contributing to significant reductions in the health cost of bone fracture treatment^(36)^.

It is important to consider the selection of the fortification vehicle within the legal framework. Food fortification can either be mandatory, designed to address certain inadequate intakes and their health consequences, or voluntary, which depends on the food manufacturer’s and consumer’s demand^(4,17)^. Mandatory food fortification programmes require minimal behaviour change and, if well implemented, can be a cost-effective public health intervention^(17)^. The food vehicle selected for a food fortification policy should be consumed regularly by the target population and ideally be industrially produced to facilitate effective fortification^(17,37)^. A review that assessed food fortification in seventy-two countries showed that most countries (*n* 55, 76 %) had mandatory wheat flour fortification with at least one nutrient and that mandatory maize (*n* 11, 15 %) or rice flour fortification (*n* 6, 8 %) with at least one nutrient was less common^(38)^. Argentina has had mandatory wheat flour fortification with iron, folate, riboflavin, niacin and thiamine since 2002, a policy designed to reach the whole population^(39)^. A pre- and post-fortification study showed reductions in Neural Tube Defects attributed to mandatory folate fortification of wheat flour^(40)^. The study shows that the prevalence of anencephaly, bifid spine and encephalocele per 10 000 births decreased between pre- and post-fortification periods from 6·92 (5·80–8·20) to 2·33 (1·99–2·72), from 8·16 (6·94–9·54) to 4·34 (3·86–4·85) and from 2·12 (1·52–2·87) to 0·73 (0·54–0·95), respectively^(40)^.

Argentina has no mandatory calcium fortification of wheat flour; however, the country has regulations for voluntary calcium fortification of staple foods^(17)^. This regulation allows for fortification of 20–50 % of the recommended nutrient intake, which represents 200–500 mg of elemental calcium per food serving. Despite these regulations, the industry does not fortify wheat flour with calcium voluntarily^(17)^. Dairy products are voluntarily fortified with calcium in Argentina; however, the high levels of low calcium intake indicate that these products are insufficient to reach the recommended calcium intake levels.

The existing laws of wheat flour fortification in Argentina would facilitate the incorporation of calcium fortification, as mills and wheat flour production infrastructure are already equipped to include micronutrients into the premix^(39)^. According to Argentina’s 2005 Food and Household survey, the main intake of flour is in the form of bread and pasta. The survey also shows that lower-income quintiles have higher flour intake and lower dairy product intake when compared with higher-income quintiles. Given this discrepancy, large-scale flour fortification would benefit the lowest quintile (poorest) which consume less calcium-rich products such as dairy products^(41)^. Wheat flour seems to be an appropriate vehicle for this population; as it is widely consumed in Argentina, it was reported by most individuals of all age groups and country regions^(4)^. Our simulations show that wheat flour could be a good and effective fortification food vehicle to improve calcium intake in Argentina and could help decrease the high prevalence of low calcium intake without placing the risk of calcium intake excess. These results show that wheat flour intake increased up to the age of 14 to less than 19 years and then decreased with age. Despite having higher flour intake, those aged 9 to less than 19 years also have the highest recommended calcium intake (EAR = 1100 mg of calcium a day), part of the reason why greater impacts on reducing low calcium intake were not observed in this group.

The information from this simulation is the first step towards designing a fortification strategy, as it assesses current calcium intake levels, wheat flour intake and the theoretical impact of different calcium fortification levels in all population age groups. This, together with the existing legal framework that allows food fortification with calcium and the current legislation of mandatory wheat flour fortification with other minerals, could facilitate a policy change from voluntary to mandatory calcium fortification. The final food fortification level will depend on several criteria, including industrial feasibility and organoleptic acceptability of wheat flour food derivatives such as bread and pasta. Future studies should assess the industrial feasibility of fortifying flour at higher levels such as 400 or 500 mg of elemental calcium per 100 g of flour. Assessments of the physicochemical and rheological characteristic of flour and flour-derived products such as bread, cakes and pastas should be performed. The available infrastructure, capacities for food processing and production systems, cost and access to calcium salts and other industrial inputs such as changes in labels and monitoring regulations need to be analysed^(4,37)^.

The population organoleptic acceptability of calcium fortification should be assessed before deciding the fortification levels and should continue once the fortification is implemented as this would ensure adherence and success of the proposed intervention^(4)^. Previous experiences usually refer to the use of calcium carbonate to fortify wheat flour; however, depending on the type of product, other salts such as calcium citrate, calcium phosphate and calcium lactate could be selected^(42,43)^.

Monitoring and evaluation strategies should be designed and implemented as part of a food fortification programme. Regulatory monitoring should ensure flour production and end-product fortification processes work effectively. Wheat and its main derivative, flour, are domestically produced on a large scale in Argentina, which is the eleventh biggest wheat producer and exporter worldwide^(44)^. Monitoring strategy implementation is feasible as 88 % of wheat is grown in three provinces of Argentina and more than half of the flour is produced and commercialised by eleven mills^(45)^.

Regular population surveys should be administered to monitor food consumption, especially the amount and type of flour consumed and calcium-rich food intake, as their changes could impact the result of the fortification strategy^(46)^. Population surveys should also assess health outcomes in the population, mainly bone health and hypertensive disorders. National and regional maternal mortality and its causes such as hypertensive disorders of pregnancy are reported annually by the Ministry of Health in Argentina^(47)^. To better monitor the impact of the fortification strategy, additional vital statistics could include incidence of pre-eclampsia, eclampsia, preterm birth as well as death and health loss from osteoporotic fractures.

Future research is needed to understand the possible interactions of fortification with multiple nutrients on iron and calcium status and assess the magnitude of the interaction to inform multiple nutrient fortification^(48)^. Short-term studies and single-meal studies have assessed that calcium from fortified foods or supplements inhibits the bioavailability of iron; however, this constraint is still under debate^(3,4,8)^. A recent systematic review suggests that this short-term effect does not translate to a long-term detriment of iron status^(49)^. Several food fortification programmes around the world include iron and calcium simultaneously, like that of the UK which mandates the fortification of wheat flour with iron, calcium and B-complex vitamins^(9)^.

### Conclusion

Considering the magnitude of calcium intake inadequacy in Argentina and the widespread intake of wheat flour, calcium-fortified flour could be further explored to improve calcium intake. Subnational simulations could be performed to identify groups that might not be reached by this strategy. As previous experiences have shown cost-effectiveness of wheat flour fortification with calcium in some countries, this strategy could be explored in Argentina, a country with high prevalence of low calcium intake and high wheat flour consumption. The analysis presented here could be used to advocate for a strategy to fortify wheat flour.

## Data Availability

The data presented in this study are openly available in http://www.extensioncbc.com.ar/wp-content/uploads/ENNyS-2007.pdf.

## References

[1] Shkembi B & Huppertz T (2021) Calcium absorption from food products: food matrix effects. Nutrients 14, 180. 35011055 10.3390/nu14010180 PMC8746734

[2] Matikainen N , Pekkarinen T , Ryhänen EM et al. (2021) Physiology of calcium homeostasis. Endocrinol Metab Clin North Am 50, 575–590. 34774235 10.1016/j.ecl.2021.07.005

[3] Cormick G & Belizán JM (2019) Calcium intake and health. Nutrients 11, 1606. 31311164 10.3390/nu11071606 PMC6683260

[4] Allen L , de Benoist B , Dary O et al. (2006) Guidelines on Food Fortification With Micronutrients. Geneva: WHO.

[5] Institute of Medicine (2011) DRI Dietary Reference Intakes Calcium Vitamin D. Washington, DC: The National Academies Press. 21796828

[6] Kumssa DB , Joy EJM , Ander EL et al. (2015) Dietary calcium and zinc deficiency risks are decreasing but remain prevalent. Sci Rep 5, 10974. 26098577 10.1038/srep10974 PMC4476434

[7] Balk EM , Adam GP , Langberg VN et al. (2017) Global dietary calcium intake among adults: a systematic review. Osteoporos Int 28, 3315–3324. 29026938 10.1007/s00198-017-4230-x PMC5684325

[8] Bourassa MW , Abrams SA , Belizán JM et al. (2022) Interventions to improve calcium intake through foods in populations with low intake. Ann N Y Acad Sci 1511, 40–58. 35103316 10.1111/nyas.14743 PMC9306636

[9] Palacios C , Cormick G , Hofmeyr GJ et al. (2021) Calcium-fortified foods in public health programs: considerations for implementation. Ann N Y Acad Sci 1485, 3–21. 32986887 10.1111/nyas.14495 PMC7891425

[10] Gomes F , Ashorn P , Askari S et al. (2022) Calcium supplementation for the prevention of hypertensive disorders of pregnancy: current evidence and programmatic considerations. Ann N Y Acad Sci 1510, 52–67. 35000200 10.1111/nyas.14733 PMC9306576

[11] Karrar SA & Hong PL (2022) Preeclampsia. Treasure Island (FL): StatPearls Publishing. 34033373

[12] Belizán JM , Gibbons L & Cormick G (2021) Maternal mortality reduction: a need to focus actions on the prevention of hypertensive disorders of pregnancy. Int J Equity Health 20, 1–6. 34454497 10.1186/s12939-021-01535-x PMC8403409

[13] Kinshella MW , Sarr C , Sandhu A et al. (2022) Calcium for pre-eclampsia prevention: a systematic review and network meta-analysis to guide personalised antenatal care. BJOG An Int J Obstet Gynaecol 129, 1833–1843. 10.1111/1471-0528.17222 35596262

[14] UNICEF (2019) World Bank Group and the United Nations Population Division. Vol. Trends in Maternal Mortality 2000–2017: Estimates by WHO. 2. Washington, D.C: World Bank Group.

[15] Knight F , Ferguson EL , Rana ZH et al. (2023) Including calcium-fortified water or flour in modeled diets based on local foods could improve calcium intake for women, adolescent girls, and young children in Bangladesh, Uganda, and Guatemala. Ann N Y Acad Sci 1526, 84–98. 37391187 10.1111/nyas.15032

[16] WHO (2020) WHO Recommendation: Calcium Supplementation During Pregnancy for the Prevention of Pre-Eclampsia and Its Complications. Geneva: WHO. 30629391

[17] Cormick G , Betrán AP , Metz F et al. (2020) Regulatory and policy-related aspects of calcium fortification of foods. implications for implementing national strategies of calcium fortification. Nutrients 12, 1022. 32276435 10.3390/nu12041022 PMC7230677

[18] Cormick G , Betran AP , Romero IB et al. (2021) Impact of flour fortification with calcium on calcium intake: a simulation study in seven countries. Ann N Y Acad Sci 1493, 59–74. 33432622 10.1111/nyas.14550 PMC9290501

[19] Cormick G , Gibbons L & Belizán JM (2022) Impact of water fortification with calcium on calcium intake in different countries: a simulation study. Public Health Nutr 25, 344–357. 32744224 10.1017/S1368980020002232 PMC8883601

[20] Johnson-Down L , L’Abbé MR , Lee NS et al. (2003) Appropriate calcium fortification of the food supply presents a challenge. J Nutr 133, 2232–2238. 12840185 10.1093/jn/133.7.2232

[21] Cormick G , Romero IB , Matamoros N et al. (2023) Calcium concentration of drinking water to improve calcium intake: a simulation study. Ann N Y Acad Sci 1524, 97–104. 37026582 10.1111/nyas.14986

[22] Ministerio de Salud de la Nación Argentina (2021) Base de Datos de la 2° Encuesta Nacional de Nutrición y Salud (ENNyS2) 2018–2019. Argentina: Ministerio de Salud de la Nación Argentina.

[23] Ministerio de Salud de la Nación Argentina (2018) Sistema de Análisis de Registro de Alimentos (SARA). Argentina: Ministerio de Salud de la Nación Argentina.

[24] U.S. Department of Agriculture & Agricultural Research Service (2011) USDA National Nutrient Database for Standard Reference, Release 24. Database 2011. Maryland: U.S. Department of Agriculture, Agricultural Research Service.

[25] Universidad Nacional de Luján (2011) Tabla de Composición de Alimentos de la Unlu. Luján: Universidad Nacional de Luján.

[26] Public Health England (2015) Composition of Foods Integrated Dataset (CoFID). London: Public Health England.

[27] Iowa State University (1995–2015) Software for Intake Distribution Estimation; Intake Monitoring Assessment and Planning Program (IMAPP). Ames: Iowa State University of Science and Technology.

[28] Government of UK (1998) The Bread and Flour Regulations 1998. London: The Stationery Office.

[29] EFSA (2015) Scientific opinion on dietary reference values for calcium. EFSA J 13, 1–82. 10.2903/j.efsa.2015.4185 PMC13151608 42109813

[30] Fulgoni VL , Keast DR , Bailey RL et al. (2011) Foods, fortificants, and supplements: where do Americans get their nutrients?. J Nutr 14, 1847–1854. 10.3945/jn.111.142257 PMC3174857 21865568

[31] EFSA Panel on Dietetic Products, Nutrition and Allergies (2012) Scientific opinion on the tolerable upper intake level of calcium. EFSA J 10, 2814. 10.2903/j.efsa.2012.2814 PMC13093087 42016128

[32] Ministerio de Salud de la Nacion (2022) Manual del Nutricionista. ENNyS 2. Buenos Aires: Ministerio de Salud.

[33] Wallace TC , McBurney M & Fulgoni VL (2014) Multivitamin/mineral supplement contribution to micronutrient intakes in the United States, 2007–2010. J Am Coll Nutr 33, 94–102. 24724766 10.1080/07315724.2013.846806

[34] Hoy MK & Goldman JD (2012) Calcium Intake of the U.S. Population, What We Eat Am NHANES 2009–2010. Beltsville (MD): United States Department of Agriculture (USDA). 36913510

[35] Scientific Advisory Committee on Nutrition (2012) Nutritional Implications of Repealing the UK Bread and Flour Regulations. London: Scientific Advisory Committee on Nutrition.

[36] Sandmann A , Amling M , Barvencik F et al. (2017) Economic evaluation of vitamin D and calcium food fortification for fracture prevention in Germany. Public Health Nutr 20, 1874–1883. 26568196 10.1017/S1368980015003171 PMC10261623

[37] Olson R , Gavin-Smith B , Ferraboschi C et al. (2021) Food fortification: the advantages, disadvantages and lessons from sight and life programs. Nutrients 13, 1118. 33805305 10.3390/nu13041118 PMC8066912

[38] Marks KJ , Luthringer CL , Ruth LJ et al. (2018) Review of grain fortification legislation, standards, and monitoring documents. Glob Heal Sci Pract 6, 356–371. 10.9745/GHSP-D-17-00427 PMC6024620 29959275

[39] Argentina HCDLN (2022) Ley 25.630. Buenos Aires: Congreso de la Nación Argentina.

[40] Sargiotto C , Bidondo MP , Liascovich R et al. (2015) Descriptive study on neural tube defects in Argentina. Birth Defects Res Part A Clin Mol Teratol 103, 509–516. 10.1002/bdra.23372 25855266

[41] Zapata ME , Rovirosa A & Carmuega E (2019) Urbano y rural: diferencias en la alimentación de los hogares argentinos según nivel de ingreso y área de residencia. Salud Colect 15, e2201. 31829400 10.18294/sc.2019.2201

[42] Department for Environment Food and Rural Affairs (2013) Bread and Flour Regulations 1998 Consultation. London: Department for Environment Food and Rural Affairs.

[43] Osler M & Heitmann BL (1998) Food patterns, flour fortification, and intakes of calcium and vitamin D: a longitudinal study of Danish adults. J Epidemiol Community Health 52, 161–165. 9616420 10.1136/jech.52.3.161 PMC1756689

[44] FAO (2022) World Food and Agriculture – Statistical Yearbook 2022. Rome: FAO.

[45] Mapa de la molienda de trigo en Argentina. Bolsa de Comercio de Rosario. https://www.bcr.com.ar/es/print/pdf/node/89589 (accessed July 2024).

[46] Food Standards Agency (2022) Amending the Bread and Flour Regulations 1998 and the Bread and Flour Regulations (Northern Ireland) 1998. London: Food Standards Agency.

[47] Ministerio de Salud de la Nación (2022) Indicadores Básicos. Argentina: Ministerio de Salud de la Nación.

[48] Ahmed A , Anjum FM , Randhawa MA et al. (2012) Effect of multiple fortification on the bioavailability of minerals in wheat meal bread. J Food Sci Technol 49, 737–744. 24293693 10.1007/s13197-010-0224-9 PMC3550827

[49] Abioye AI , Okuneye TA , Odesanya AMO et al. (2021) Calcium intake and iron status in human studies: a systematic review and dose-response meta-analysis of randomized trials and crossover studies. J Nutr 151, 1084–1101. 33758936 10.1093/jn/nxaa437

